# Laparoscopic-assisted total gastrectomy for early gastric cancer with situs inversus totalis: report of a first case

**DOI:** 10.1186/s12893-015-0059-4

**Published:** 2015-06-19

**Authors:** Mamoru Morimoto, Tetsushi Hayakawa, Hidehiko Kitagami, Moritsugu Tanaka, Yoichi Matsuo, Hiromitsu Takeyama

**Affiliations:** Department of Surgery, Kariya Toyota General Hospital, Kariya, Japan; Department of Gastroenterological Surgery, Nagoya City University Graduate School of Medical Sciences, Kawasumi 1, Mizuho-cho, Mizuhoku, Nagoya, 467-8601 Japan

**Keywords:** Situs inversus totalis, Gastric cancer, Laparoscopic-assisted total gastrectomy

## Abstract

**Background:**

Situs inversus totalis is a relatively rare condition and is an autosomal recessive congenital defect in which an abdominal and/or thoracic organ is positioned as a “mirror image” of the normal position in the sagittal plane. We report our experience of laparoscopic-assisted total gastrectomy with lymph node dissection performed for gastric cancer in a patient with situs inversus totalis.

**Case presentation:**

A 58-year-old male was diagnosed with cT1bN0N0 gastric cancer. There were no vascular anomalies on abdominal angiographic computed tomography with three-dimensional reconstruction. laparoscopic-assisted total gastrectomy was performed with D1+ lymph node dissection, in accordance with the Japanese Gastric Cancer Treatment Guidelines. There were no intraoperative issues, and no postoperative complications.

**Conclusions:**

This was the first report describing laparoscopic-assisted total gastrectomy with the standard typical lymph node dissection in the English literature. We emphasize that the position of trocars and the standing side of the primary surgeon during the lymph node dissection are critical.

## Background

Situs inversus totalis (SIT) is a relatively rare condition found in only approximately 1 per 5,000 to 20,000 people [1]. SIT is an autosomal recessive congenital defect in which an abdominal and/or thoracic organ is positioned as a “mirror image” of the normal position in the sagittal plane. Laparoscopic surgery for such patients has been reported upon, with most reports describing laparoscopic cholecystectomy [1]. Reports of advanced laparoscopic surgery in such patients are also increasing in accord with the overall progress of laparoscopic procedures. In 2003, the first case of laparoscopic surgery for a SIT patient with gastric cancer was reported [2]. Only a few cases of laparoscopic assisted distal gastrectomy (LADG) for gastric cancer have been reported [2–8]. However, there are no reports of laparoscopic assisted total gastrectomy (LATG) for gastric cancer in patients with SIT in the English literature. We herein report a case of LATG with lymph node dissection and esophagojejunostomy using the overlap method in a patient with early gastric cancer and SIT.

## Case presentation

**Fig. 1:**
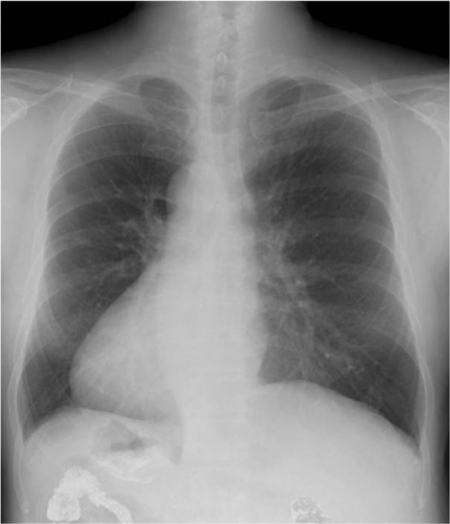
Dextrocardia was evident on chest radiography from the frontal view

**Fig. 2:**
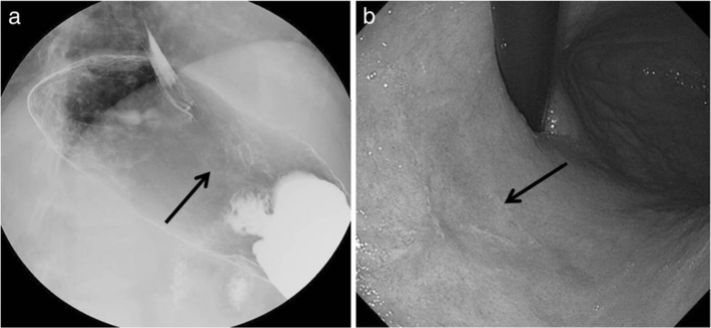
**a**, **b** Upper gastrointestinal endoscopy and gastrointestinal imaging study showed a superficial lesion with a slight depression of the upper gastric body

**Fig. 3:**
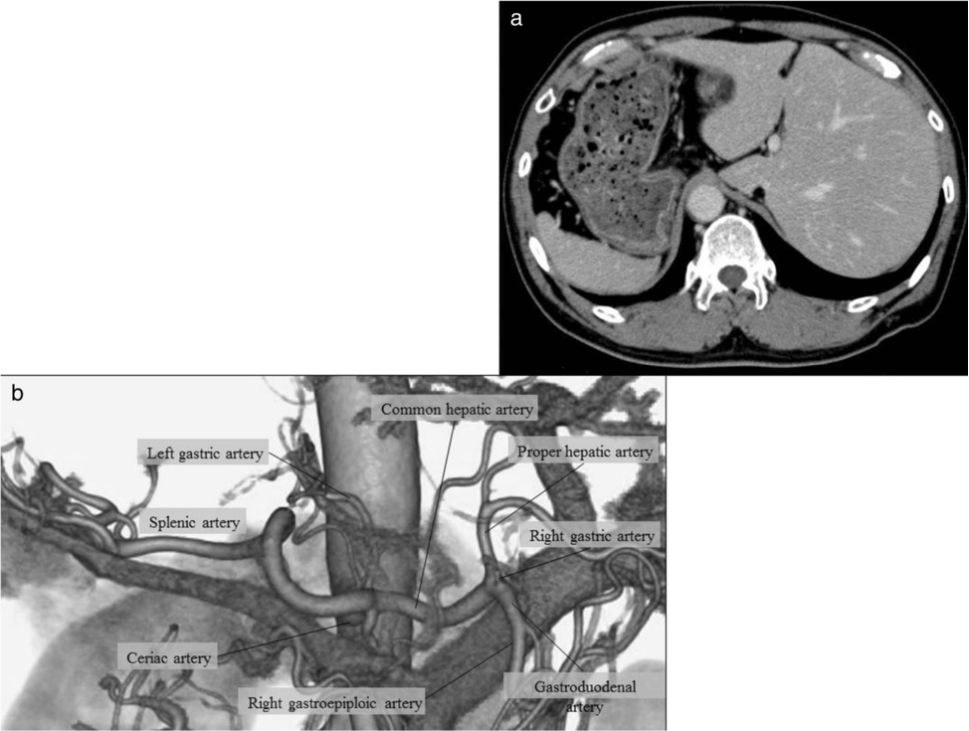
**a** Enhanced abdominal CT showed transposition of the abdominal organs and identified no metastasis to the lymph nodes or to the distant organs. **b** Three-dimensional reconstruction image of CT angiography showed no vascular anomalies

A 58-year-old male with SIT was diagnosed with early gastric cancer by an esophagogastroduodenoscopy (EGD) performed at an outside hospital, and was subsequently referred to our hospital for further evaluation and surgical treatment. He was diagnosed with SIT 5 years previously. He had been healthy until this diagnosis with no other underlying disease or any family history of SIT or gastric cancer. History and physical examination revealed right orchidoptosis. Laboratory examination, including tumor markers, showed no abnormal findings. Electrocardiogram (ECG) examination revealed right bundle branch block. Chest x-ray suggested dextrocardia (Fig. 1). Upper gastrointestinal imaging (UGI) and repeat EGD identified a superficial lesion with slight depression (0-IIc) measuring 20 × 25 mm in size on the posterior side of the lesser curvature of the upper gastric body (Fig. 2a, b). The lesion was 3 cm distal to the esophagogastric junction (EGJ). Biopsy revealed poorly differentiated adenocarcinoma. Abdominal computed tomography (CT) showed that all intraabdominal organs were inversely positioned (Fig. 3a). Prior to surgery, abdominal angiographic CT with 3D reconstruction was performed to reveal any other anatomic variations and to verify the locations of the major vasculature. There were no arterial variations (Fig. 3b). Chest and abdominal CT did not reveal any metastases, including none to the liver or lung. Based on findings of the biopsy, UGI, EGD and CT, a 0-IIc lesion of the upper gastric body, clinical stage cT1b, cN0cH0cP0cM0, stage IB according to the Japanese Classification of Gastric Carcinoma was diagnosed [9]. We decided to perform LATG with standard lymph node dissection (D1 + No. 7, 8a, 9) in accordance with the Japanese Gastric Cancer Treatment Guidelines [10].

**Fig. 4:**
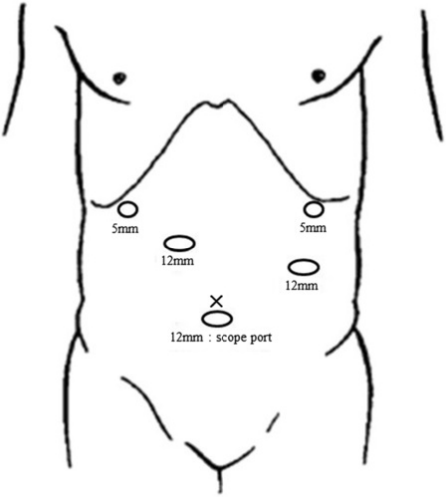
Placement of ports

**Fig. 5:**
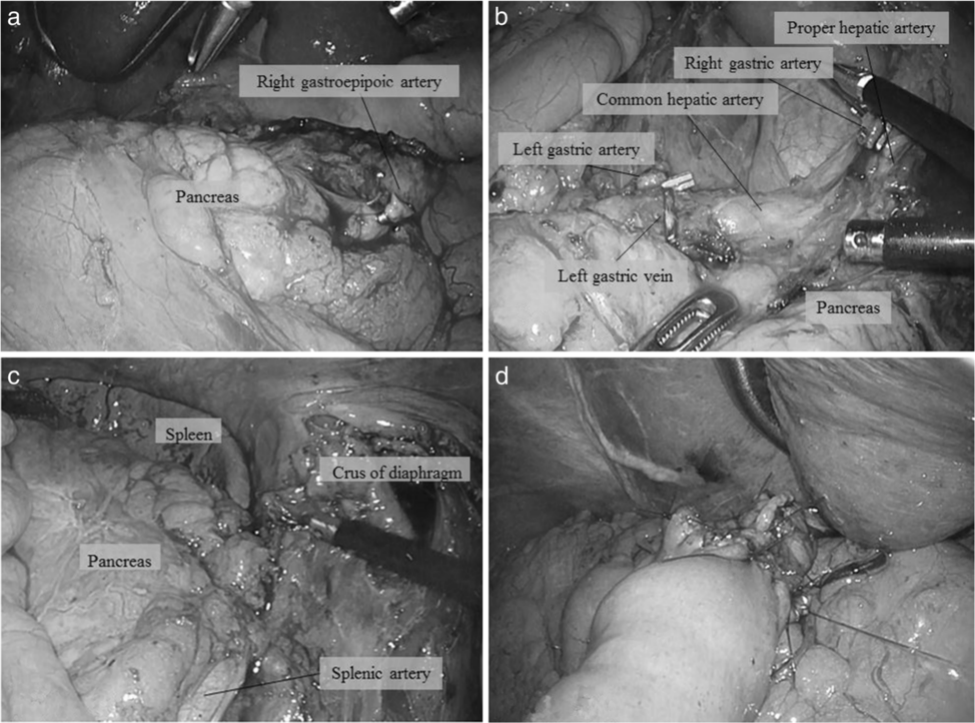
**a** Dissection of lymph nodes in lymph node basin 6 (infrapyloric lymph nodes). **b** Dissection of lymph nodes in lymph node basins 8a, 9, and 7 (along the common hepatic artery, the celiac artery, and the left gastric artery). **c** Dissection of lymph node basin 11p (along the splenic artery). **d** Reconstruction via esophagojejunostomy using the overlap method

After general anesthesia was induced, the patient was placed in a supine position. The position of trocars is showed in Fig. 4. First, a 12-mm trocar was placed in the umbilical site, and carbon dioxide was injected into the peritoneal cavity at 10 mmHg. A laparoscope was inserted into the abdomen through the 12-mm trocar. An additional 4 ports were placed in the left and right subcostal positions and lateral abdominal regions. The surgeon was positioned on the right side of the patient for dissection of lymph node basins 5, 6, 7, 8a, and 9 and was positioned on the left side for the dissection of lymph node basins 1, 2, 3, 4sa, 4sb, 4d, and 11p. At the beginning of the operation, the surgeon was positioned on the left side of the patient and the greater omentum was dissected. The location of the spleen was confirmed, and the 4sa node basin (along the short gastric vessels) and 4sb node basin (along the left gastroepiploic vessels) were dissected. The left gastroepiploic vessels were clipped and divided. Next, the surgeon was positioned on the right side of the patient, and the node basin 6 (infrapyloric lymph nodes) was dissected (Fig. 5a) and the duodenum was transected with a linear stapling device intraperitoneally. Next, the node basins 5 (suprapyloric lymph nodes), 8a [lymph node along the common hepatic artery (CHA)], 9 [lymph node around the celiac artery (CA)], and 7 [lymph node along the left gastric artery (LGA)] were dissected safely (Fig. 5b). Next, the surgeon was positioned on the left side of the patient again, and the node basins 11p [along the proximal splenic artery (SA)], 1 (right pericardial lymph node), 2 (left pericardial lymph node), and 3 (along the lesser curvature) were dissected completely in accord with Gastric Cancer Treatment Guidelines [10] (Fig. 5c). We exposed the abdominal esophagus and transected it at an appropriate resection line using an endoscopic linear stapler. The resected stomach and surrounding fatty tissue including harvested lymph nodes were placed in a plastic specimen bag. The specimen in the bag was retrieved through an extended umbilical port incision. At last, we performed an esophagojejunostomy using the overlap method intraperitoneally (Fig. 5d). Operating time was 359 min, and blood loss was 90 mL. The final pathology showed a poorly differentiated 0-IIc lesion with invasion limited to the mucosa. There was no metastasis in any of the retrieved lymph nodes. The final stage was pT1aN0M0, Stage 1A according to the Japanese Classification of Gastric Carcinoma [9]. There were no immediate postoperative complications, and the patient was discharged 7 days after the operation. The patient is still alive without recurrence or symptoms 4 years after surgery.

## Discussion

In this case, we made two important clinical observations. First, 3D reconstruction of an abdominal CT angiography image should be done to examine a blood stream in detail in SIT patients preoperatively. Second, the position of trocars and the standing side of the primary surgeon during the dissection of basins 5, 7, 8a, and 9 are critical.

SIT is a rare congenital anomaly. SIT can be accompanied by cardiopulmonary malformations, familial long QT syndrome, total esophageal duplication, agnathia, and a variety of urologic anomalies. Typically, the frequency of cardiovascular anomalies is 10 times greater than in a patient with normal anatomy [6]. In other report, SIT is associated with cardiovascular malformations in 8 % of cases [11]. Therefore, preoperative identification of any abnormal vasculature is important, because abnormal vasculature carries with it the risk of misidentifying anatomy and causing unanticipated injury of important vessels. In our case, major vascular abnormalities were not found. However, it is required to examine a blood stream in 3D reconstruction of an abdominal CT angiography image preoperatively.

Second, the position of trocars and the standing side of the primary surgeon during the dissection of basins 5, 7, 8a, and 9 are critical. Laparoscopic surgery has been performed for SIT patients in numerous case reports, including laparoscopic cholecystectomy [12], laparoscopic colectomy [13], laparoscopic fundoplication [14], and laparoscopic gastric band surgery [15]. In 2003, LADG for gastric cancer in a patient with SIT was successfully performed in Japan, and was the first reported case of its kind [2]. Furthermore, in 2010, LADG with D1 + β lymph-node dissection for early gastric cancer was successfully performed in Japan [3]. It was the first case of laparoscopic gastrectomy with extended lymph node dissection. The reported cases of laparoscopic gastrectomy for gastric cancer with SIT are listed in Table 1 [2–8]. LATG is not performed as frequently as LADG even in patients without SIT due to the technical difficulty of the laparoscopic approach, particularly the esophagojejunostomy and complete lymph node dissection. The present patient was the first report describing LATG with the standard typical lymph node dissection (D1+) [10] in the English literature. It is a very important element where the surgeon inserts the trocar, and which direction the surgeon operates on from. However, there is no consensus about it until now, because LATG has never been reported for the patients with SIT. Thus, based on our experience, we describe the position of trocars and the standing side of the primary surgeon when the surgeon performs LATG for the patients with SIT.

**Table 1:** Previous reports of laparoscopic surgery for gastric cancer with SIT

No.	Author	Age	Sex	Anomalies of blood vessels	Operation	Lymoh node dissection^*^	Position of surgeon	Operating time (min)	Blood loss (ml)	Stage^**^	Post operation
1	Yamaguchi (2003)	76	M	ND	LADG	ND	ND	ND	ND	ND	ND
2	Futawatari (2010)	53	M	no anomalies	LADG	D1 + β	left side (opposite the usual side)	300	350	IA	no complications
3	Seo (2011)	60	M	no anomalies	LADG	D1 + β	right side (same the usual side)	200	70	IB	no complications
4	Kim (2012)	47	M	no anomalies	RADG	D1 + β	same the usual side	300	ND	IIIB	no complications
5	Fujikawa (2013)	60	F	no anomalies	LADG	ND	opposite the usual side	234	5	IB	mechanical obstruction (re-operation)
6	Min (2013)	52	M	the CHA from SMA 2 branches from the LGA	LADG	D1+	same the usual side	220	100	IB	no complications
7		68	M	no anomalies	LADG	D1+	same the usual side	117	50	IA	no complications
8	Sumi (2014)	42	M	the LHA from the SMA	LADG	D1 + No.7, 8a, 9	opposite the usual side	313	90	IB	no complications
9	Our case	58	M	no anomalies	LATG	D1+	opposite the usual side	359	90	IA	no complications
							(except for no.5, 7,8a, 9)			IA	no complications

^*^According to Japanese gastric cancer treatment guidelines 2010 (ver. 3)

^**^According to Japanese classification of gastric carcinoma: 3rd English edition

The surgeon should perform the dissection of basins 5, 7, 8a, 9 from right side of patient same as normal operation. We did not perform the dissection of basins 5, 7, 8a, and 9 from the left side of patient as the surgeon’s right hand (dominant hand) using the ultrasonic cut and coagulation instrument can contact the proper hepatic artery (PHA).

This risk is not completely avoided with the surgeon on the right side, however. The ultrasonic cut and coagulation instrument may hit the PHA perpendicularly, and damage the vessels during dissection of basin 5. However, lymph node dissection from the right side of the patient is safer than that from the left side of the patient if we pay attention to the cavitation of the ultrasonic cut and coagulation instrument so as not to damage the vessels.

**Fig. 6:**
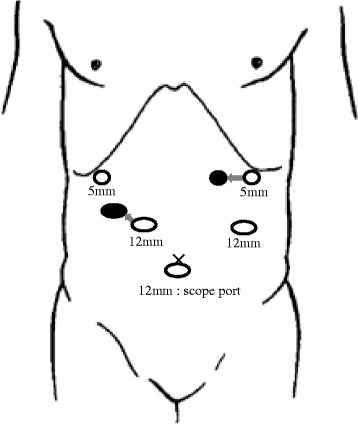
Optimum positions of the trocars for LATG with SIT

Furthermore, in normal operation, if the surgeon is right-handed, he inserts the lower right trocar more vertically up and towards the center. That is because he can dissect node basins 7, 8a and 9 precisely and more deeply, and he can reach the spleen using right hand for dissecting lymph node around the splenic artery. In SIT, there is no need to insert the lower right trocar toward the center because there is no spleen in the left side of abdomen. However, the trocar should be inserted in the higher position, because it is difficult to perform the lymph node dissection around the CHA as the pancreas interferes with movement of the right hand. We describe the procedure after the surgeon moves to the left side of the patient. The surgeon should insert the upper left trocar (which is used with the right hand) more toward the center. As a result, the surgeon’s hand can reach the spleen and he can avoid the injury of PHA during the dissection of node basins 11p. The diagram of the optimum positions of the trocars in LATG with SIT is shown in Fig. 6.

There are 4 cases in the literature describing the operation done with the surgeon standing in the standard position [4, 5, 7]. One of these authors reported that the mirror image led to confusion during the operation [4]. Furthermore, another of these authors reported that even slight confusion of the anatomy can jeopardize the patient’s life, and that only a surgeon experienced with laparoscopic gastrectomy should perform the operation [7]. There is a report of the use of surgical instruments in the non-dominant hand from the opposite side of patient when operating on patients with SIT [6]. We advise against this technique to prevent the damage of major vessels and to ensure adequate node dissection. However, robot-assisted distal gastrectomy (RADG) is an exception to this as the surgeon does not need to change his positon during surgery because of the centered robotic view of the field with easy changing of the instruments between hands [5].

Our procedure of LATG with D1+ dissection and esophagojejunostomy using the overlap method was completed in 359 min with 90 mL blood loss. In other reports of LADG, the median operating time and blood loss were 267 min and 90 mL, respectively. It is logical that total gastrectomy has a longer operative time given the more extensive dissection necessary compared to distal gastrectomy. Also, the esophagojejunostomy using overlap method was carried out from the left side of the patient, we could perform it safely. As in our procedure, lymph node dissection and esophagojejunostomy can be performed safely and efficiently by coordinating the position of trocars, and alternating the standing side of the primary surgeon over the course of the case.

## Conclusions

In conclusion, there has been marked improvement of the surgical technique and instruments used in laparoscopic surgery recently. Since the LATG was performed at our hospital more than 200 times, the operating team has become very comfortable with performing it. While performing LATG with SIT, we have encountered some problems present. Thus, we have described the important technical aspects of LATG with lymph node dissection for patients with gastric cancer and SIT, namely recognition of the anatomy, with particular attention to the vasculature as determined by preoperative 3D reconstruction of an abdominal CT angiography image. Furthermore, it was important to simulate the position of the trocar insertion, and the change of the position of the primary surgeon preoperatively.

## Consent

Written informed consent was obtained from the patient for publication of this Case report and any accompanying images. A copy of the written consent is available for review by the Editor of this journal.

## Abbreviations

SIT: Situs inversus totalis
LATG: Laparoscopic-assisted total gastrectomy
3D: Three-dimensional
LADG: Laparoscopic-assisted distal gastrectomy
EGD: Esophagogastroduodenoscopy
ECG: Electrocardiogram
UGI: Upper gastrointestinal imaging
EGJ: Esophagogastric junction
CT: Computed tomography
CHA: Common hepatic artery
CA: Celiac artery
LGA: Left gastric artery
SA: Splenic artery
PHA: Proper hepatic artery
RADG: Robot-assisted distal gastrectomy
SMA: Superior mesenteric artery
LHA: Left hepatic artery
DG: Distal gastrectomy
TG: Total gastrectomy
PG: Proximal gastrectomy
ND: Not described
